# The efficacy of adding hyperthermia to the treatment of advanced NSCLC patients based on the states of EGFR

**DOI:** 10.18632/aging.204148

**Published:** 2022-06-28

**Authors:** Yanhua Zhou, Qiulu Zhong, Dongning Huang, Li Qin, Jian Huang, Chunhui Wang, Binglin Chen, Menghua Chen, Yihe Li, Wenqi Liu

**Affiliations:** 1 Department of Radiation Oncology, The Second Affiliated Hospital of Guangxi Medical University, Nanning 530000, Guangxi, China; 2 Department of Oncology, The Liuzhou Worker’s Hospital, Liuzhou 545000, Guangxi, China

**Keywords:** non-small-cell lung carcinoma, epidermal growth factor receptor, hyperthermia, first-line treatment, posterior-line therapy

## Abstract

Background: The study aims to explore the efficacy of adding hyperthermia to the treatment of advanced NSCLC patients based on the states of epidermal growth factor receptor (EGFR).

Patients and methods: We included 205 advanced NSCLC patients who were received hyperthermia plus other treatment (hyperthermia group) or non- hyperthermia and other treatments (non- hyperthermia group). The OS and progression free survival (PFS) were retrospectively estimated. Using Kaplan-Meier and the log-rank test compare the OS and PFS between the groups.

Results: The median follow-up was 22 months. The Univariate analysis have shown that 1-year OS and PFS_first_ rates in the hyperthermia group and non- hyperthermia group were 83.3% vs 71.5% (*P*=0.010) and 62.0% vs 42.7% (*P*=0.001). The subgroup analyses revealed that patients didn’t have EGFR mutant who received hyperthermia had significantly higher 1 year OS and PFS_first_ rates than those treated with non- hyperthermia (OS: 79.1% vs 65.2% *P*=0.037, PFS: 64.2% vs 36.5%, *P*=0.001). For patients with EGFR mutation, there was no significant difference between the two groups. The PFS_first_ in first-line and PFS_post_ in posterior-line was no significant difference between the groups.

Conclusions: This retrospective study revealed that adding hyperthermia to the treatment of NSCLC patients without EGFR mutation had better prognosis than those who did not adding hyperthermia to the regimen. Moreover, adding hyperthermia in first-line or in posterior-line treatment was no significant difference. However, these results need more prospective studies to confirm the conclusions.

## INTRODUCTION

Non-small-cell lung carcinoma (NSCLC) is the most common type of lung cancer (LC), which accounts for 85% of all lung cancer cases [1–3]. Surgery is the prior treatment for early-stage NSCLC patients. However, nearly 75% patients are in advanced stages when they are first diagnosed. The National Comprehensive Cancer Network (NCCN) [4] guideline recommend tyrosine-kinase inhibitor (TKI) therapy to patients with EGFR mutation. All NSCLCs today are divided according to EGFR status positive and negative as shown by EGFR and polymorphisms having significance [5–7]. Immunotherapy (IO) is applied for EGFR negatives. Several studies, such as KEYNOTE 024, IMpower110, EMPOWER-Lung1, KEYNOTE189, KEYNOTE 407, have shown that IO not only could prolong the PFS, but also could improve the OS [8–12]. However, patients with EGFR mutation who treated with IO had limited efficacy. Previous meta-analysis study showed that IO did not benefit patients with EGFR mutation, but increased adverse reactions [13]. And the latest phase II single arm trial had been ended early because of lacking significant clinical efficacy [14]. The efficacy of adding IO to the treatment of patients with EGFR mutation in advanced NSCLC is still controversial.

Whether IO should be added to the regimen of advanced NSCLC with EGFR mutated remains unclear. It is also unclear why adding IO to advanced NSCLC with EGFR mutated decreases the efficacy of treatment. It may be related to the immune microenvironment. Hyperthermia is an effective measure to overcome tumor hypoxic cells, and it mainly kills the S phase cells, which were resistant to radiotherapy. Moreover, it can reduced the production of immunosuppressive cells. Guo et al. [15] found that hyperthermia could transform Treg cells into Th17 cells by increasing IL-6 levels. Wendt MK [16] found hyperthermia could also indirectly reduce TGF-β by up-regulating the expression of miR-10b to inhibit breast cancer cell proliferation. Guo et al. [17] found hyperthermia could not only activate Cytotoxic T lymphocyte (CTL) by increasing the expression of B7 molecules and ICAM-1, but it could also increase CD+4 /CD+8 ratio. Therefore, hyperthermia can improve the tumor microenvironment (TME), and promote the efficacy of IO in advanced NSCLC.

On the basis of these theories, we hypothesis that patients with EGFR mutation may not benefit from hyperthermia. Our study aims to explore the prognosis of adding hyperthermia to the treatment of advanced NSCLC with different EGFR mutated states and evaluate the efficacy of adding hyperthermia as the first-line treatment method or as a posterior-line method.

## RESULTS

### Characteristics of patients

The median follow-up was 22 months. A total of 205 patients were included the study. Among the 205 III-IV NSCLC patients, 77 patients in the hyperthermia group and 128 patients in the non- hyperthermia group. The baseline characteristics of the two groups were shown in Table 1. There were 162(79.1%) cases of adenocarcinoma (AC) and 43(20.9%) cases of squamous cell carcinoma (SCC); among them, there were 63(30.7%) patients in stage III, whereas 142(69.3%) patients in stage IV. There were 69(33.7%) patients with EGFR mutation and 136(66.3%) patients in EGFR wild-type or undetected genes or other mutated gene.

**Table 1:** Baseline characteristics of hyperthermia group and non- hyperthermia group.

**Characteristic**	**Hyperthermia group (*N*=77)**	**Non- hyperthermia group (*N*=128)**	***P* value**
**Gender**			0.419
Male	53(68.8%)	81(63.3%)	
Female	24(31.2%)	47(36.7%)	
**Age**			0.812
<60	35(45.5%)	56(43.7%)	
≥60	42(54.5%)	72(56.3%)	
**Smoke**			0.302
YES	32(41.6%)	44(34.4%)	
NO	45(58.4%)	84(65.6%)	
**ECOG score**			0.137
≤1	75(97.4%)	117(91.4%)	
>1	2(2.6%)	11(8.6%)	
**Pathology**			0.376
adenocarcinoma	58(75.3%)	104(81.3%)	
squamous carcinoma	19(24.7%)	24(18.7%)	
**Stage**			0.175
III	28(36.4%)	35(27.3%)	
IV	49(63.6%)	93(72.7%)	
**EGFR mutation state**			0.879
EGFR mutation	25(32.5%)	44(34.4%)	
EGFR no mutation or others	52(67.5%)	84(65.6%)	
**Treatment**			0.286
combined treatment without EGFR-TKI	55(71.4%)	81(63.3%)	
combined treatment with EGFR-TKI	22(28.6%)	47(36.7%)	

### Failure patients

Up to September 2021, there were 29 patients (14.1%) lost to follow-up. Twenty-six patients (33.8%) died, 16 patients (20.8%) encountered recurrences, 14 patients (18.2%) occurred distant metastasis and 7 patients (9.1%) encountered recurrences and distant metastasis in hyperthermia group. In terms of non- hyperthermia group, 81 (63.3%)of 128 patients died,52(40.6%) of 128 patients were relapsed, 16(12.5%) of 128 patients had distant metastasis and 24(18.8%) of 128 patients encountered recurrences and distant metastasis (Table 2).

**Table 2:** Failure patients of two group.

	**No. of patients(%)**
**Pattern of failures**	**Hyperthermia group (77)**	**Non- hyperthermia group (128)**
Died	26(33.8%)	81(63.3%)
Relapsed	16(20.8%)	52(40.6%)
Distant metastasis	14(18.2%)	16(12.5%)
Relapsed and distant metastasis	7(9.1%)	24(18.8%)

### The outcomes of survival

**Figure 1:**
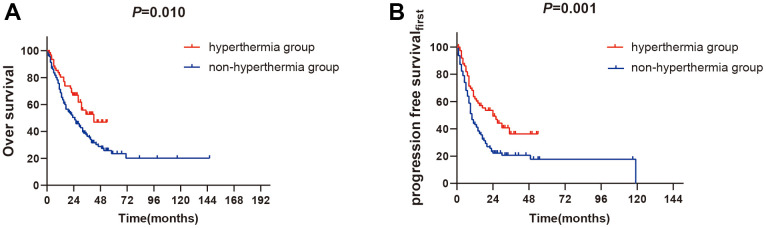
**The outcomes of survival between hyperthermia group and non-hyperthermia group.** (**A**) 1-year OS rates between two groups (*P<0.05*). (**B**) 1-year PFS_first_ rates between two groups (*P<0.01*).

The 1-year OS and PFS_first_ rates of hyperthermia group and non- hyperthermia group were 83.3% versus 71.5% (*P*=0.010, Figure 1A) and 62.0% versus 42.7% (*P*=0.001, Figure 1B).

### Subgroups analysis

**Figure 2:**
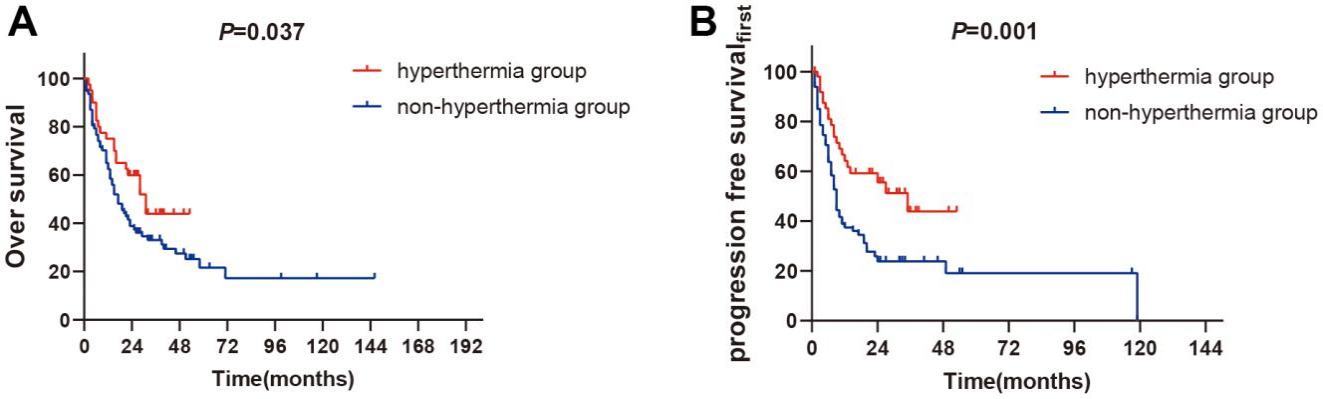
**The subgroups analysis of non-EGFR mutation between two groups.** (**A**) 1-year OS rates of non-EGFR mutation between two groups (P<0.05). (**B**) 1-year PFS_first_ rates of non-EGFR mutation between two groups (*P<0.01*).

The subgroups analyses have shown that the 1-year OS rate in hyperthermia group and in non- hyperthermia group was 79.1% versus 65.2% (*P*=0.037, Figure 2A) in the non- EGFR mutant subset, while the 1-year PFS_first_ rate in hyperthermia group and non- hyperthermia group were 64.2% versus 36.5% (*P*=0.001, Figure 2B). The outcomes had significant difference between the two groups.

**Figure 3:**
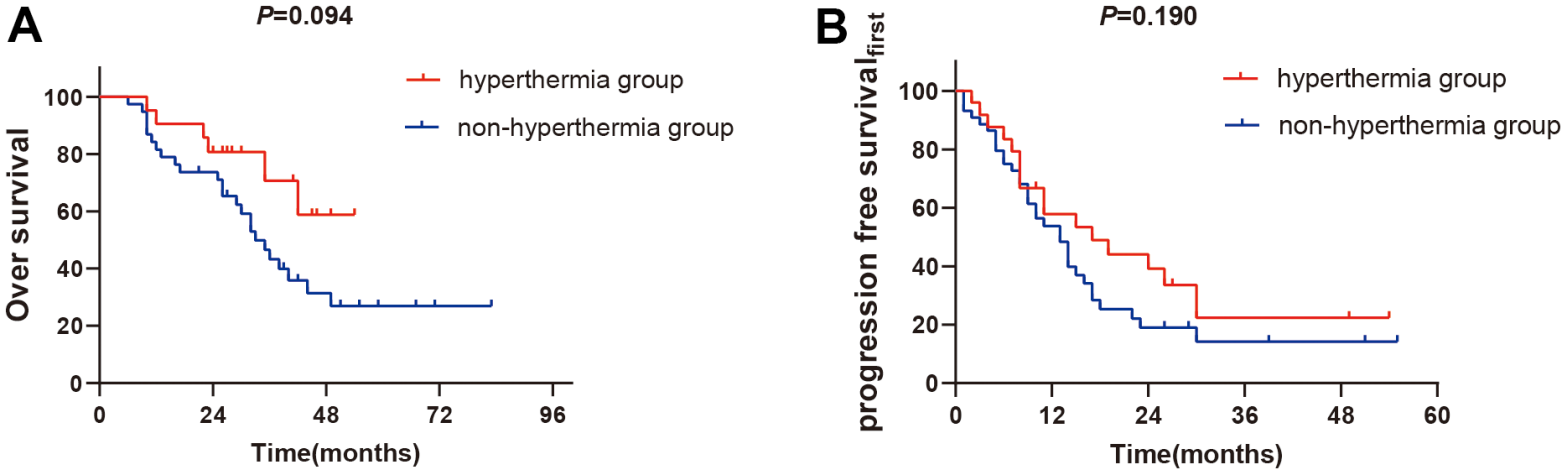
**The subgroups analysis of EGFR mutation between two groups.** (**A**) 1-year OS rates of EGFR mutation between two groups (P>0.05). (**B**) 1-year PFS_first_ rates of EGFR mutation between two groups (*P>0.05*).

For patients with EGFR mutation, the 1-year OS and PFS_first_ rates did not significantly differ between the two groups (the 1-year OS rate: 91.7% vs. 83.5%, *P*=0.094, Figure 3A. the 1-year PFS_first_ rate: 57.9% vs. 53.8%, *P*=0.190, Figure 3B).

### Prognostic factors

Multivariate Cox regression analysis demonstrated that smoke, clinical stage and hyperthermia were the independent prognostic factors for PFS_first_ and OS (Tables 3, 4).

**Table 3:** Multivariate analysis of progression-free survival.

**Variable**	**Hazard ration**	**95% CI**	***P* value**
Gender	1.039	0.648-1.666	0.875
Age	0.863	0.601-1.238	0.423
Smoke	0.497	0.312-0.790	0.003
Pathology	1.116	0.684-1.820	0.660
Stage	0.355	0.226-0.557	0.000
ECOG score	1.084	0.494-2.382	0.840
Hyperthermia	1.940	1.291-2.914	0.001
Target Therapy	1.481	0.880-2.491	0.139
Radiotherapy	0.974	0.668-1.421	0.892
Chemotherapy	0.963	0.608-1.526	0.873
EGFR mutation state	0.739	0.469-1.162	0.190

**Table 4:** Multivariate analysis of overall survival.

**Variable**	**Hazard ration**	**95% CI**	***P* value**
Gender	0.752	0.448-1.263	0.281
Age	1.121	0.755-1.666	0.571
Smoke	0.583	0.359-0.947	0.029
Pathology	0.997	0.589-1.688	0.992
Stage	0.395	0.244-0.640	0.000
ECOG score	0.523	0.275-0.995	0.048
hyperthermia	1.671	1.036-2.693	0.035
Targeted	1.838	1.004-3.366	0.048
Radiotherapy	1.135	0.736-1.748	0.567
Chemotherapy	2.108	1.314-3.383	0.002
EGFR mutation state	1.706	0.986-2.954	0.056

### Posterior-line analysis

**Figure 4:**
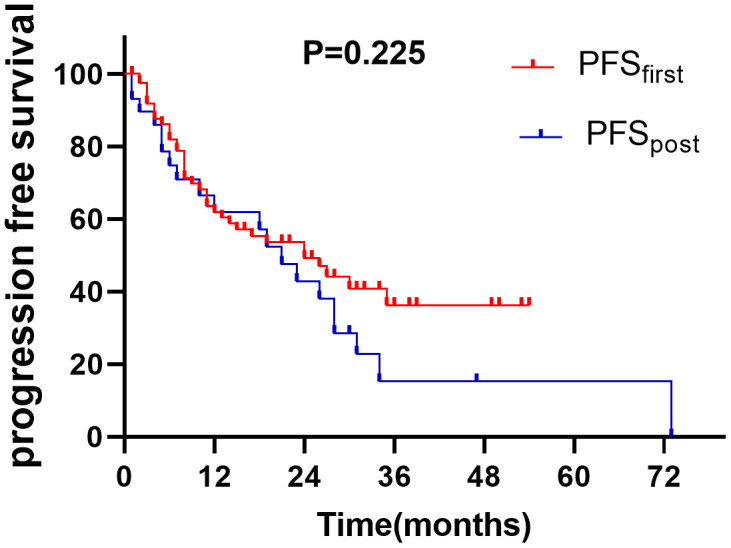
The PFS of adding hyperthermia in first-line and posterior-line treatment (*P>0.05*).

Of patients who occurred progress and had not received hyperthermia in first-line treatment, twenty-nine patients were treated with hyperthermia as a posterior-line treatment method. Of the 29 patients, 12(41.4%) relapsed, 2 (6.9%) had distant metastasis and 6(20.7%) both had recurrences and distant metastasis. The median PFS were no significant difference between the first-line treatment and posterior-line treatment (24m VS 21m, *P* = 0.225, Figure 4).

## DISCUSSION

The relationship between tumor stroma cells is very important and that certain cytokines play an important role in the relationship between tumor hosts [18]. This study compared the efficacy of adding hyperthermia to the current regimens or without hyperthermia in advanced NSCLC. Our results showed that patients who received hyperthermia had better PFS_first_ and OS than those without hyperthermia. A retrospective study [19] showed that hyperthermia combined with RT had a better prognosis and did not increase side effects. The author found the objective response rate (ORR) and 3-year OS rates between two groups were 97% versus 70% and 37% versus 6.7%, retrospectively (*P*<0.05). Italian scholars [20] were the first to report that hyperthermia combined with Chemoradiotherapy in LC in 1998, the results demonstrated that stage IV patients who received hyperthermia had longer OS (13.2m vs 8.4m, *P*<0.01). However, the simple size was small and these studies were retrospective studies. A review showed hyperthermia could exert synergistic effect with other treatments [21], Which were similar to our study. The mechanisms of hyperthermia enhance the anticancer effects of radiotherapy are as below: 1. DNA double-strand breaks (DSB) are the most critical radiation-induced damage, which is the main cause of cell death. It can inhibit the repair of DSB in NSCLC cell line, promotes apoptosis and inhibits the invasion of tumor cells when radiotherapy combined with hyperthermia [22]. 2. Hyperthermia can ameliorate the TME such as low PH, hypoxia, and vascular immaturity through increases blood flow and perfusion [23]. 3. Cells in the S phase which are insensitive to radiotherapy, are sensitive to hyperthermia. Hyperthermia increases the effectiveness of chemotherapy by increasing the concentration of chemotherapy drugs, inhibiting DNA repair, increasing the release of free radicals, reversing the drug resistance of chemotherapy drugs and so on [24].

The subgroup analyses in our study revealed that patients didn’t have EGFR mutant who received hyperthermia had significantly higher 1 year OS and PFS_first_ rates than those treated with non- hyperthermia (*P*=0.001). And for patients with EGFR mutation, there was no significant difference between the two groups. The results were consistent with our expected results. Microwave ablation (MWA) as another method of Heat therapies has been widely used in many malignant tumors [25–27]. Wei et al. [28] conducted a retrospective study on 61 NSCLC patients who were known EGFR status treated MWA combined with chemotherapy / EGFR-TKI. The results showed that the median PFS of EGFR mutant and wild-type patients were 8.3 months and 5.4 months respectively (*P* = 0.162), and the median OS were 17.8 months and 27.2 months (*P* = 0.209). The results showed that patients with EGFR mutation could not benefit from MWA, our findings were consistent with them. However, the sample size of that study was small and only compared the effects of MWA combine with other treatments based on different EGFR status. Therefore, they [29] further performed on 58 EGFR mutant patients, who treated with TKI with or without MWA. The results revealed that no significant difference in objective response rate (ORR), PFS and OS between the two groups (*P*=0.230, 0.640 and 0.288 respectively). As a result, they suggested that MWA should not be recommended for unselected patients with EGFR-sensitive mutations. Our study further explored the efficacy of adding hyperthermia for NSCLC patients with different EGFR states and our outcomes are consistent with the results of the above studies.

The reason why patients with EGFR mutation could not benefit from hyperthermia now is still unclear. We suppose hyperthermia had a positive effect on immune microenvironment as we described previously, and the special immune microenvironment in patients with EGFR mutation. Dong et al. [30] performed a pool-analysis of 3283 patients from 15 studies to systematically assessed the association between EGFR mutation and PD-L1 expression. They found that EGFR wild-type tumors were more likely to be PD-L1-positive than EGFR mutant tumors (OR: 1.79; 95% CI 1.10–2.93; *P* = 0.02). They analyzed the protein and mRNA profiles of PD-L1 in the repository (The Cancer Genome Atlas; TCGA) and internal (Guangdong Lung Cancer Institute; GLCI) databases and performed IHC detection of PD-L1 in resected NSCLC tissues found same results. What’s more, they explored the correlation between EGFR status and CD8+ T-cell infiltration. Analysis of mRNA profiles in the GLCI cohort indicated that patients with EGFR mutations had a lower CD8A expression than those with EGFR wild-type (*P* = 0.031). IHC analysis of CD8+ TILs in the 255 resected NSCLC specimens confirmed that EGFR mutant tumors showed lesser T-cell infiltration than EGFR wild-type ones (*P* = 0.003). They also found patients with sensitive EGFR mutation display low immunogenicity and show impaired response to PD-1 blockade. Eri Sugiyama et al. [31] have shown that tumor mutation burden (TMB) was decreased and regulatory T cells (Treg) was increased in EGFR-mutated lung adenocarcinomas (LUADs). Thus, we suspected low TMB, low tumor infiltrating lymphocytes (TILs) and high Treg in patients with EGFR mutation may resulted in poor efficacy of IO of them. Hyperthermia can promote the efficacy of IO in advanced NSCLC by improving the TME, which may lead to patients with EGFR mutation do not benefit from hyperthermia. But more basic researches should be carried out to clarify the mechanisms.

Moreover, our study also investigated the best time to add hyperthermia. The outcomes demonstrated that the PFS_first_ in first-line and PFS_post_ in posterior-line was no significant difference between the entire groups. Takayuki Ohguri et al. [32] carried out a study to assess the efficacy of re-irradiation plus regional hyperthermia for 33 recurrent NSCLC patients, the results showed that the median disease PFS after re-irradiation were 6.7 months. In contrast, our median PFS_post_ was 21 months.

However, our study was a retrospective clinical study. The sample size was small and the follow-up time was short. Some patients did not detect EGFR or combined with other gene mutations may impact on the results. It need more prospectively, large sample size, randomized controlled trails to confirm the results.

## CONCLUSIONS

This retrospective study revealed that adding hyperthermia to the treatment of NSCLC patients without EGFR mutation had better prognosis than those who didn’t adding hyperthermia to the regimen. Moreover, adding hyperthermia in first-line or in posterior-line treatment was no significant difference. However, these results need more prospective studies to confirm the conclusions.

## MATERIALS AND METHODS

### Patients

We retrospectively collected patients from the Second Affiliated Hospital of Guangxi Medical University and Liuzhou Worker’s Hospital between January 2018 and December 2019. All patients had pathologically confirmed adenocarcinoma (AC) and squamous cell carcinoma (SCC) of lung and stage III-IV disease by the staging criteria of the 8^th^ edition of the American Joint Committee on Cancer Staging System (AJCC) and the International Union Against Cancer (UICC) [33]. The inclusion criteria were as follows: 1) Patients aged 18 to 70 years old, 2) ECOG score ≤ 2, 3) Patients with complete clinical data, 4) Stage III -IV disease restaged by the 8^th^ edition of AJCC/UICC TNM staging system, 5) Patients previously didn’t received any treatment, 6) Patients didn’t have any malignancies before. Exclusion criteria were: 1) ECOG score ≥3, 2) Patients with severe hepatic, renal and pulmonary dysfunction, 3) The pathological type was not adenocarcinoma or squamous cell carcinoma,4) Cognitive dysfunction. A total of 205 patients were included in this study based on the inclusion and exclusion criteria. On the basis of adding hyperthermia or not to the regimen in the advanced NSCLC before first progression, we divided all the eligible patients into hyperthermia group (*N*= 77) and non- hyperthermia group (*N* = 128). There were 25(32.5%) patients had EGFR mutation in hyperthermia group while 44(34.4%) patients in non- hyperthermia group among the groups. The patients’ base-line characteristics were shown in Table 1.

### Treatment

### Surgery

Lobectomy or partial or total pneumonectomy± lymph node dissection through thoracotomy or thoracoscopy.

### Chemotherapy

Chemotherapy schemes were as follows: cisplatin or carboplatin + pemetrexed (AP), gemcitabine + cisplatin or carboplatin (GP or GC), docetaxel or paclitaxel or albumin bound paclitaxel 2+ cisplatin or carboplatin (TP), repeated every three weeks, 4-6 cycles.

### Radiotherapy (RT)

All included patients were received 6 MV-X ray liner accelerator intensity modulated radiation therapy (IMRT), and the treatment steps were as follows: (1) Patients were positioned and fixed, scanned and positioned with simulated CT (Philips, Brilliance, Netherlands and Siemens, AS Definition Open20, Germany) and the scanning layer spacing was 3mm. (2) Target delineation: We used Treatment planning system (TPS) 13.6 (Varian, Vitalbeam, USA) and Raystaion4.7.5 (Varian, Trilogy, USA and Elekta, Precise, UK) to delineate the target volumes. According to ICRU Report No. 83, the target area included: ① gross tumor volume (GTV): the range of primary lesions determined according to imaging, including primary lung lesions (GTV) and regional lymph node metastasis (GTVnd). ② Clinical tumor volume (CTV): it included GTV, subclinical lesions and areas that may be invaded by tumors. ③ Planning tumor volume (PTV) was the range determined according to organ movement and daily positioning error to ensure the treatment dose. ④ Dangerous organs: dangerous organs included: left lung, right lung, double lungs, heart, trachea, esophagus, brain stem, spinal cord, optic nerve, optic chiasm, etc. 3) According to the NCCN guidelines, the prescription dose and organ endangering limit were given, and the plan was evaluated: ① prescription dose: radical radiotherapy for lung lesions: PGTV: 60gy-66Gy/30-33 f, PTV1: 50-54Gy/ 30-33 fractions at 5 fractions per week during a period of 6-7 weeks. SBRT:60Gy/10f. Palliative radiotherapy for lung lesions: PGTV: 45-50Gy, PTV1: 36-40Gy/25-27 fractions. Other lesions such as whole brain radiotherapy, vertebral body and bone: PTV: 30Gy / 10f or 40Gy/20f. ② Limit of Organ at risk (OAR): the maximum dose of brain stem was less than 54Gy; The maximum dose of spinal cord < 45Gy; The maximum dose of optic nerve and optic chiasm < 50Gy; Single lung V5 ≤ 65%, V20 ≤ 30%, V30 ≤ 20%; Heart V30 ≤ 30%, average cardiac irradiation dose ≤ 35Gy; Esophageal V50 ≤ 50%, average dose ≤ 34 Gy. The maximum dose of trachea was less than 60 Gy.

### Target therapy

(1) Oral Gefitinib 250mg / time, once a day, (2) Erlotinib 150mg / time, once a day, (3) Ektinib 125mg / time, three times a day, (4) Ositinib 80mg / time, once a day, (5) Afatinib 40mg / time, once a day, (6) Kezotinib 250mg / time, twice a day.

### Hyperthermia

One hour before other treatment, Jilin Maida medical radiofrequency hyperthermia system NRL-004 was used to patients for 60 minutes, the temperature was controlled at 42° C, twice a week, with an average of 4-8 times.

### Endpoints

The primary endpoint of this study was 1-year PFS. PFS_first_ defined as the time from the diagnosis of the disease to the first progression or death from any cause. PFS_post_ in the Posterior line therapy adding hyperthermia was defined as from disease first progression to the last progression or death from any cause. The secondary endpoint was 1-year OS, which defined as the time from disease diagnosis to death or the last follow-up.

### Statistical analysis

SPSS 22.0 software (IBM) and graphpad prism 8 software were used to analyze the data. Chi-square test was used for continuous variables, and t-test was used for categorical variables. Kaplan-Meier method was conducted for the analysis of the time-to-event endpoints, and the log-rank test was used to compare the differences between the two groups. Multivariate analysis were performed in the detection of prognostic factors related to the endpoints. Using graphpad prism 8 to draw the survival curve. All statistical tests were two-sided, and P<0.05 was considered statistically significant.

### Ethics approval

The study was approved by the ethics committees of The Second Affiliated Hospital of Guangxi Medical University, and The Liuzhou Worker’s Hospital.
